# Transcriptomic signatures in brain and blood related to cognitive and psychiatric phenotypes of Prader–Willi syndrome

**DOI:** 10.1038/s41598-025-33041-3

**Published:** 2025-12-24

**Authors:** Shokouh Shahrokhi, Emma K. Baker, Michael See, Mirana Ramialison, Dinusha Gamage, Fernando J. Rossello, Helen Heussler, Michael Duhig, Robert D. Nicholls, Anthony J. Hannan, Melissa C. Southey, Olivia Veatch, Waheeda Hossain, Minh Bui, David J. Amor, Merlin G. Butler, David E. Godler

**Affiliations:** 1 Department of Paediatrics, University of Melbourne, Parkville, VIC Australia; 2 Murdoch Children’s Research Institute, Parkville, VIC Australia; 3 School of Psychology and Public Health, La Trobe University, Bundoora, VIC Australia; 4 reNEW Novo Nordisk Foundation Centre for Stem Cell Medicine & Stem Cell Biology, Murdoch Children’s Research Institute, Parkville, VIC Australia; 5 Australian Regenerative Medicine Institute, Monash University, Clayton, VIC Australia; 6 Monash Bioinformatics Platform, Monash University, Clayton, VIC Australia; 7 Department of Clinical Pathology, University of Melbourne, Parkville, VIC Australia; 8 Centre for Clinical Trials in Rare Neurodevelopmental Disorders, Children’s Health Queensland Hospital and Health Service, South Brisbane, Brisbane, QLD Australia; 9 Centre for Child Health Research, Faculty of Medicine, University of Queensland, South Brisbane, Brisbane, QLD Australia; 10 Division of Genetic and Genomic Medicine, Department of Pediatrics, UPMC Children’s Hospital of Pittsburgh and University of Pittsburgh, Pittsburgh, PA USA; 11 Florey Institute of Neuroscience and Mental Health, Parkville, VIC Australia; 12 Department of Anatomy and Physiology, School of Biomedical Sciences, University of Melbourne, Parkville, VIC Australia; 13 Precision Medicine, School of Clinical Science at Monash Health, Monash University, Clayton, VIC Australia; 14 Cancer Epidemiology Division, Cancer Council Victoria, Melbourne, VIC Australia; 15 Department of Psychiatry and Behavioural Sciences, University of Kansas Medical Centre, Kansas City, KS USA; 16 Centre for Epidemiology and Biostatistics, Melbourne School of Population and Global Health, University of Melbourne, Carlton, VIC Australia; 17 The Murdoch Children’s Research Institute, 50 Flemington Rd, Parkville, VIC 3052 Australia

**Keywords:** *RPS18*, Prader-Willi syndrome, Behavioral issues, Intellectual functioning, Brain transcriptomics, Biomarkers, Diseases, Genetics, Neurology, Neuroscience

## Abstract

This study defined gene expression changes across different cell types in prefrontal cortex (PFC) of donors with Prader-Willi syndrome (PWS) compared to controls and examined relationships between these changes in blood and PWS symptoms. 8,338 long non-coding RNAs and 17,079 protein-coding genes were examined using single-nucleus RNA-sequencing (snRNA-seq) in the PFC of 8 donors with PWS (4 deletion, 4 non-deletion), and 4 age- and sex-matched neurotypical controls. snRNA-seq analyses showed an increased proportion of interneurons in both PWS groups compared to controls. Fifty-four genes and related pathways were consistently dysregulated across all cell types in the PFC of the PWS group compared to controls, with *RPS18* being the only protein-coding gene upregulated in PWS PFC across all comparisons. Increase in *RPS18* mRNA levels in peripheral blood mononuclear cells (PBMCs) of another cohort (16 deletion, 20 non-deletion, ages 1–45 years) assessed using droplet digital PCR was found to be associated with intellectual functioning and challenging behaviors, but not autistic traits in children with PWS due to non-deletion (< 13 years old; *N* = 19). If confirmed in future studies, these findings may lead to development of prognostic biomarkers and therapeutics targeting consistently dysregulated genes and related pathways between brain and periphery.

**Supplementary Information:**

The online version contains supplementary material available at 10.1038/s41598-025-33041-3.

## Introduction

Prader-Willi syndrome (PWS) is the leading genetic cause of life-threatening obesity, with growth hormone deficiency and short stature, learning difficulties, autistic traits, and psychiatric problems^1–3^. Failure to thrive, hypotonia and hypogonadism are typical features in infants, with food seeking and hyperphagia emerging at ~ 5 years of age^1^. PWS is caused by loss of expression of paternally inherited genes in the 15q11-q13 region^4–6^. This can result from: (i) deletions in this region that occur in 60 to 70% of cases; (ii) maternal uniparental disomy (matUPD) that occurs in 25 to 35% of PWS cases; and (iii) imprinting center defect (ICD), epimutation or microdeletion that occur in ~ 5% of cases^1,7^. Individuals with PWS having 15q11-q13 deletions have been reported to have lower verbal IQ (VIQ) scores than those with matUPD^8^. While these genotype-phenotype observations have emerged, primarily around the different genetic subtypes of PWS, there is a lack of peripheral tissue (such as blood-based) biomarkers of prognostic utility for co-occurring conditions, especially psychiatric illness and hyperphagia. There is also a lack of understanding of underlying mechanisms linked to these co-morbidities. This study thus aimed to address these gaps by: (i) establishing a cell type-specific differential expression map in human brain tissues beyond the 15q11-q13 region; and (ii) identifying genes dysregulated consistently between cell types in blood and brain and related to severity of PWS.

## Methods and materials

### Cohort characteristics

The cohort for the brain transcriptomics studies comprised 8 deceased donors with PWS (four deletion and four non-deletion genetic subtypes) and four deceased age- and sex-matched neurotypical controls (22 to 52 years old) (Table S1). The brain tissues were provided by the National Institute of Child Health and Human Development (NICHD) Brain and Tissue Bank for Developmental Disorders, University of Maryland, Baltimore.

Molecular diagnosis of PWS for each donor was performed using *SNURF*-*SNRPN* promoter DNA methylation analysis, with etiology confirmed using real-time PCR analysis of *SNRPN* copy number, as previously^9^, as well as, SNP array genotyping, and ddPCR analysis of maternally expressed *UBE3A* mRNA (Supplementary Note S1, Fig S1). The prefrontal cortex (PFC) / Brodmann area 9 was chosen because: (i) abnormal expression of genes in PFC has been linked to the severity of other neurodevelopmental disorders including idiopathic autism^10^; and (ii) the PFC has been reported to have lower grey matter in individuals with PWS compared to matched controls, and in individuals with deletion as compared to non-deletion genetic subtype of PWS^11^.

An independent cohort of 36 individuals living with PWS (16 deletion and 20 non-deletion [17 with matUPD and 3 with ICD]) was used to examine relationships between expression of genes shortlisted from the brain transcriptomics studies in peripheral blood mononuclear cells (PBMCs) and PWS symptoms. Participants were recruited through Victorian Clinical Genetics Services (VCGS); Hunter Genetics; the PWS Clinic at the Royal Children’s Hospital (RCH), Melbourne; Genetic Services of Western Australia; the Prader-Willi Association of Australia; and the Prader-Willi Research Foundation of Australia, as previously^12^. Inclusion and exclusion criteria for these cohorts and specifics on assessments of intellectual functioning and behavioral features are provided in the Note S1.

### Procedures

Individuals with PWS included in the gene expression-phenotype studies completed developmental / intellectual functioning and behavioral assessments at the RCH or within their own home, with venous blood collected at the end of each appointment (Note S1).

### Single-nucleus RNA sequencing (snRNA-seq)

**Fig. 1:**
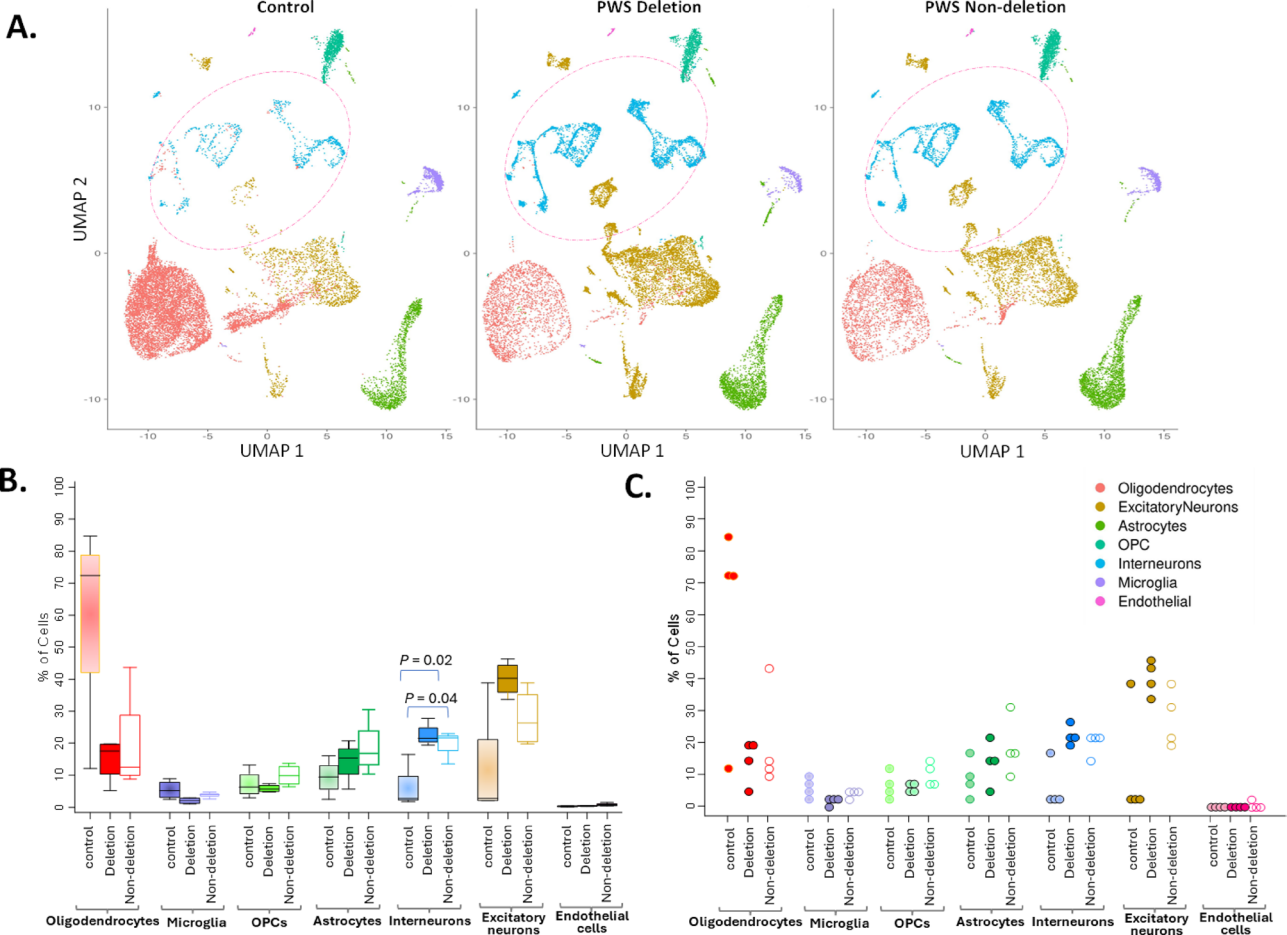
Intergroup comparisons between different cell type populations detected by 3’ single-nucleus RNA sequencing (snRNA-seq) in prefrontal cortex (Brodmann area 9) of 4 neurotypical controls, 4 PWS due to deletion subtype and 4 non-deletion subtypes. (**A**) UMAP projections of 20,000 nuclei in total per control, deletion or non-deletion PWS brain tissue group (5,000 cells per brain sample; with 4 samples per group). (**B**) boxplot and (**C**) scatter plot intergroup comparisons of percentage of cells identified as one of the 7 cell types characterized between control, deletion and non-deletion PWS subtypes. Note: Ovals with broken pink line in panel A highlight interneuron clusters present in PWS in significantly higher numbers as compared to control tissues. Kruskal–Wallis test was used for comparisons between the groups for each cell type, with median and interquartile range presented for each group in panel B.

Specifics for processing of brain samples, isolation of nuclei, quality assessments of RNA from brain tissues, and SNP analysis for demultiplexing and snRNA-seq are provided in Note S2. Sample processing, nuclei isolation, and bioinformatics were performed at the MCRI with snRNA-seq performed by the Genomics Platform of the Garvan Institute of Medical Research. Demultiplexed and processed data was generated for 8,338 long non-coding RNAs and 17,079 protein-coding genes for 7 different cell types, including microglia, astrocytes, oligodendrocytes, oligodendrocyte precursor cells (OPCs), excitatory neurons, interneurons and endothelial cells (Fig. 1 and Table S2) in the PFC of 8 donors with PWS and 4 age- and sex-matched neurotypical controls. Differential gene expression and pathway analysis were carried out for all cell types other than endothelial cells (removed due to their low numbers relative to other cell types). A pseudobulk gene matrix was created by aggregating gene counts across cells per sample for a pseudobulk analysis. Comparisons for these were then performed using the Limma package implemented in the Degust web-based tool^13^. Genes with log2 fold change greater than 1 and a false discovery rate (FDR) less than 0.05 were selected as differentially expressed genes (DEGs). The DEGs were then uploaded onto Metascape for pathway analysis^14^. The reliability of Seurat integration in removing batch effects was independently verified using Harmony, as detailed in Note S2 (Fig. S2, Data S1).

### Gene expression analysis utilizingdroplet digital PCR

Five ml of blood in EDTA tubes were collected as part of an earlier study^12^. Ficoll gradient separation was used to isolate PBMCs from blood, RNA extraction and cDNA synthesis were performed, as previously^12,15^. To establish the optimal reference gene panel for normalization of droplet digital PCR (ddPCR)-based gene expression studies, analyses on cDNA from PBMC samples of controls and individuals with PWS were performed using nine assays (*YWHAZ*,* SDHA*,* GAPDH*,* ATP5F1B*,* RPL13A*,* EIF4A2*,* TOP1*,* UBE2D2*,* GUS)* (Table S3). The ddPCR data were then analyzed using geNorm software (PrimerDesign, Ltd, Camberley, United Kingdom), as previously (Fig. S3)^15^. Specifics for ddPCR conditions and data analysis protocol used are included in Note S3.

### Data analysis

The summary statistics were presented using median and interquartile range, and the nonparametric Kruskal–Wallis test or Mann-Whitney test were used for comparisons of the groups. *RPS18* mRNA copy numbers were normalized to copy numbers from a stably expressed set of internal control genes determined using the geNorm approach on the same set of cDNA samples from PWS and control cohorts (Fig. S3). The robust regression, using M-estimator and robust standard errors, was used to assess the relationship between each phenotypic measure and *RPS18* mRNA levels. Adjustments for confounders were undertaken where appropriate. The method of least squares with robust standard error was also used when there were no outliers presented. Spearman’s rank correlation was used to assess the relationship between each PWS Behavioral Questionnaire (PWSBQ) measure and intellectual functioning and autism features scores in children as described in Note S1. False Discovery Rate (FDR) was used to adjust *P* values for multiple testing. All analyses were conducted using the software STATA (http://www.stata.com).

## Results

### snRNA-seq analyses in brain tissues

snRNA-seq analyses in the PFC showed more than a two-fold increase in the proportion of interneurons in both PWS groups compared to controls (Fig. 1). There were no statistically significant differences detected for other cell types from these intergroup comparisons. Given the high variability in oligodendrocyte proportions among control individuals, additional analyses were performed, excluding oligodendrocytes to assess whether this variability affected the results. These comparisons revealed a significant increase in the proportion of interneurons, and a decrease in OPCs and microglia when comparing the combined PWS group to controls, as well as in both deletion and non-deletion groups individually compared to controls. Additionally, the proportion of excitatory neurons was significantly higher in the deletion versus control comparison (Table S4). Between the deletion and non-deletion groups, only excitatory neurons and OPCs were different, with higher proportion of excitatory neurons and lower OPCs in the deletion group.

**Fig. 3:**
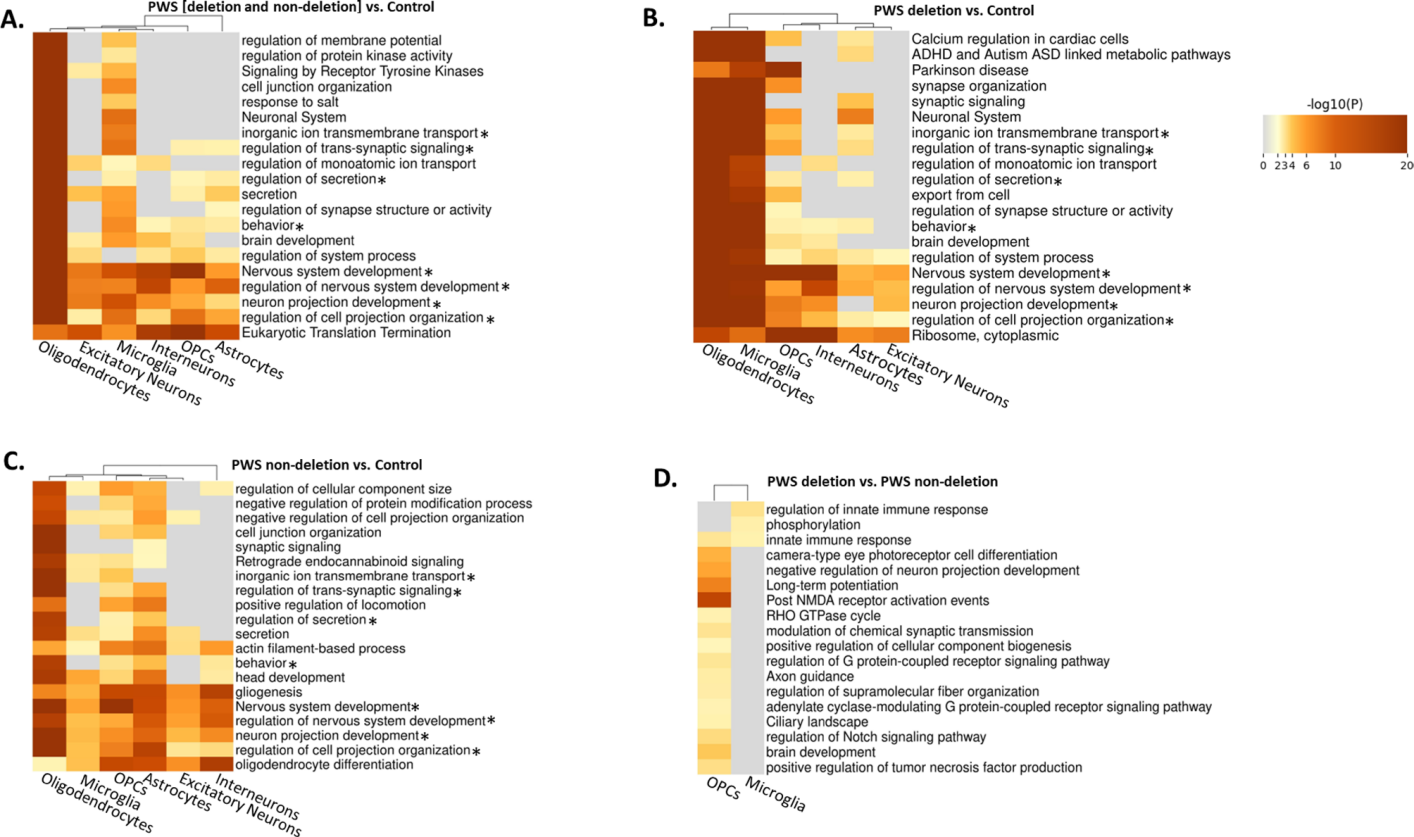
Gene Ontology enrichment analysis for the DEGs in different cell types in the prefrontal cortex between PWS due to deletion, PWS due to a non-deletion subtype and matched controls (*n* = 4 each group). Comparisons between: (**A**) combined PWS (deletion and non-deletion) and controls; (**B**) deletion PWS and controls; (**C**) non-deletion PWS and controls; (**D**) deletion and non-deletion PWS groups. *Shared pathways across multiple comparisons.

Oligodendrocytes showed the highest number of DEGs as compared to all other cell types from the combined PWS group (deletion and non-deletion) versus control comparisons (Fig. 2A). Moreover, the deletion group showed higher number of DEGs and affected Gene Ontology enrichment analytic pathways as compared to the non-deletion group, with the majority of these specific to oligodendrocytes and microglia for both comparisons (Figs. 2B and 3). Interestingly, the numbers of DEGs for microglia alone, and those shared with oligodendrocytes from deletion versus control comparisons (Fig. 2B) were also considerably higher than for non-deletion versus control comparisons (Fig. 2C), with the lowest number of DEGs observed for deletion versus non-deletion comparisons (Fig. 2D).

**Fig. 4:**
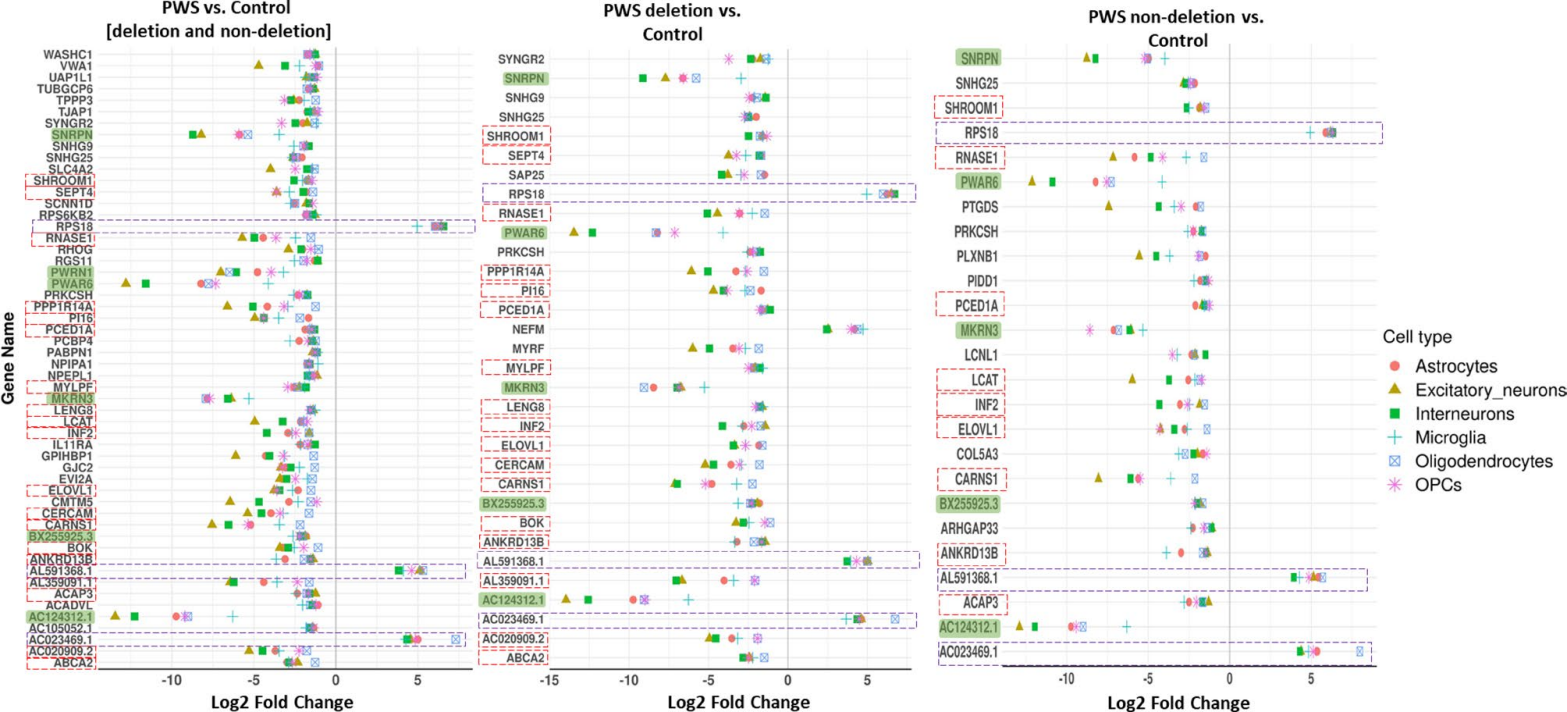
Direction and magnitude of fold change in expression of DEGs conserved between all cell types in the prefrontal cortex of PWS due to deletion, PWS due to a non-deletion as compared to matched controls (*n* = 4 each group). Note: Red rectangles with a broken line highlight DEGs located outside of the 15q11-q13 PWS-imprinted locus, downregulated in all cell types across multiple PWS versus control group comparisons. Purple rectangles with a broken line highlight DEGs located outside of the 15q11-q13 PWS-imprinted locus upregulated in all cell types across multiple PWS versus control group comparisons. Green boxes highlight DEGs located at the 15q11-q13 PWS-imprinted locus for genes expected to be silenced in all PWS subtypes, but not controls.

DEGs were then shortlisted, if consistent between all cell types, as we hypothesized that the loss of PWS-imprinted gene expression at 15q11-q13 across all cell types and tissue types in PWS, including blood^16^, would be associated with DEGs involved in counterbalancing this loss across all cell types and tissues. 54 such DEGs and pathways were identified in the PFC of donors with PWS versus controls; with 32 DEGs from deletion versus controls; and 25 from non-deletion versus control comparisons (Figs. 2 and 4). Detailed description of the results from the transcriptome-wide gene set enrichment analysis of differentially expressed gene lists identified between groups in PFC studies are provided in the Note S4. Interestingly, there were no DEG shared between all of the cell types from deletion versus non-deletion comparisons (Fig. 2D). OPCs had the highest number of DEGs for deletion versus non-deletion comparisons, with only 2 DEGs shared between more than one cell type (OPCs and microglia), which were *TUBA4A* and *PARP9*.

While most DEGs were located outside the 15q11-q13 region, as expected those imprinted in the 15q11-q13 region were silenced in all cell types and PWS genetic subtypes, but not in controls (Fig. 4). Of the DEGs located outside 15q11-q13, only 11 were consistently present across all three comparisons (Fig. 4 and Data S2). These included 2 long non-coding RNAs and 9 protein-coding genes involved in regulation of nervous and skeletal muscle systems (i.e. *SHROOM1* and *CARNS1*), as well as lipid metabolism (i.e. *PCED1A* and *ELOVL1*).

Another 10 DEGs were consistently present for the combined PWS group versus control, and deletion versus control comparisons (Fig. 4 and Data S2). These included 8 protein-coding genes and 2 long non-coding RNAs, with DEGs representing genes involved in regulation of smooth muscle function (i.e. *PPP1R14A* and *MYLPF*), cell death and Wnt signaling pathway (i.e. *SEPT4*, *PI16*, and *BOK*), as well as, function of the immune system, macrophage lipid metabolism, and neural development (i.e. *LENG8*, *ABCA2*, and *SEPT4*).

Only 2 shortlisted DEGs were consistently present for the combined PWS group versus control, and non-deletion versus control comparisons (Fig. 4 and Data S2). These included protein-coding genes, *LCAT* and *ACAP3*, involved in regulation of cholesterol transport, neuron migration, and regulation of neuron projection development.

*RPS18* was the only protein-coding gene upregulated across all PWS groups (deletion, non-deletion, and all sub-types combined) comparisons with controls (Fig. 4). It was also the only one of the 69 ribosomal DEGs (Data S3) upregulated based on snRNA-seq data in all cell types in PWS PFC and was thus chosen for follow-up gene expression (assessed using ddPCR) versus phenotype studies utilizing peripheral tissues.

For the remaining ribosomal DEGs, only three were downregulated based on snRNA-seq data. These were *RPS6KB2*,* RPS17*, and *RPS6KA4*, with *RPS6KB2* being significantly downregulated in all cell types in the combined PWS group as compared to controls. *RPS6KB2* was also significantly downregulated in the deletion versus control comparisons for OPCs, oligodendrocytes, interneurons, microglia, and astrocytes, and for OPCs, oligodendrocytes, astrocytes, and interneurons for non-deletion versus control comparisons.

### Relationships between mRNA and phenotypic measures

We examined the relationship between *RPS18* mRNA levels using ddPCR in PBMCs and PWS symptoms in the total 36 participants: nine were younger than 5 years of age, 12 were between 5 and 12 years of age, 2 adolescents at 13 and 18 years of age and 13 adults between 19 and 45 years of age. Of these, one 6-year-old female with matUPD did not have an *RPS18* mRNA value due to failed ddPCR after several repeat attempts and was thus excluded from subsequent *RPS18* mRNA levels versus phenotype studies. Another 5.4-year-old male with matUPD did not have valid standardized IQ scores due to the floor effect observed in the lower functioning range.

The remaining 26 individuals (> 5 years of age) were included in studies examining relationships between gene expression in PBMCs and PWSBQ scores (> 5-year age bracket). Of these, 11 were children (5 to 12 years: 8 with matUPD; 2 with deletion; and 1 with ICD), 2 were adolescents (one with ICD and the other with a deletion), and 13 adults (19 to 45 years: 3 with matUPD; 10 with deletion). Relationships were assessed between *RPS18* mRNA levels in PBMCs and: (i) standardized IQ scores for 19 children (4 months to 12 years of age: 11 with matUPD; 6 with deletion; and 2 with ICD); (ii) ADOS-2 scores for 17 children (4 months to 12 years of age: 11 with matUPD; 4 with deletion; and 2 with ICD) (Table 1).

**Table 1:** Univariate regression analysis assessing relationships between *RPS18* mRNA levels in PBMCs of children with PWS and intellectual functioning and autism features (< 13 years of age).

		All			Non-deletion			
	*N*	Coef	se	*P*	*N*	Coef	se	*P*
VIQ Standardized	19	-3.921	7.565	0.611	14	-13.93	10.58	0.213
PIQ Standardized	19	-17.06	7.651	0.040	14	-31.29	8.790	**0.004** ^2^
FSIQ Standardized^1^	19	-9.598	6.053	0.132	14	-21.07	7.149	**0.013** ^2^
ADOS CSS^+^	17	2.220	1.649	0.200	13	3.683	2.121	0.113
SA CSS^+^	17	1.561	1.320	0.257	13	2.001	1.740	0.277
RRB CSS	17	2.192	2.040	0.300	13	1.343	2.627	0.619

Note: Regression analyses were conducted using least squares method, unadjusted and ^1^adjusted for age; ^2^*P* value < 0.05 after adjusting for multiple testing, separately for intellectual functioning and autism features, using False Discovery Rate (FDR).

**Fig. 5:**
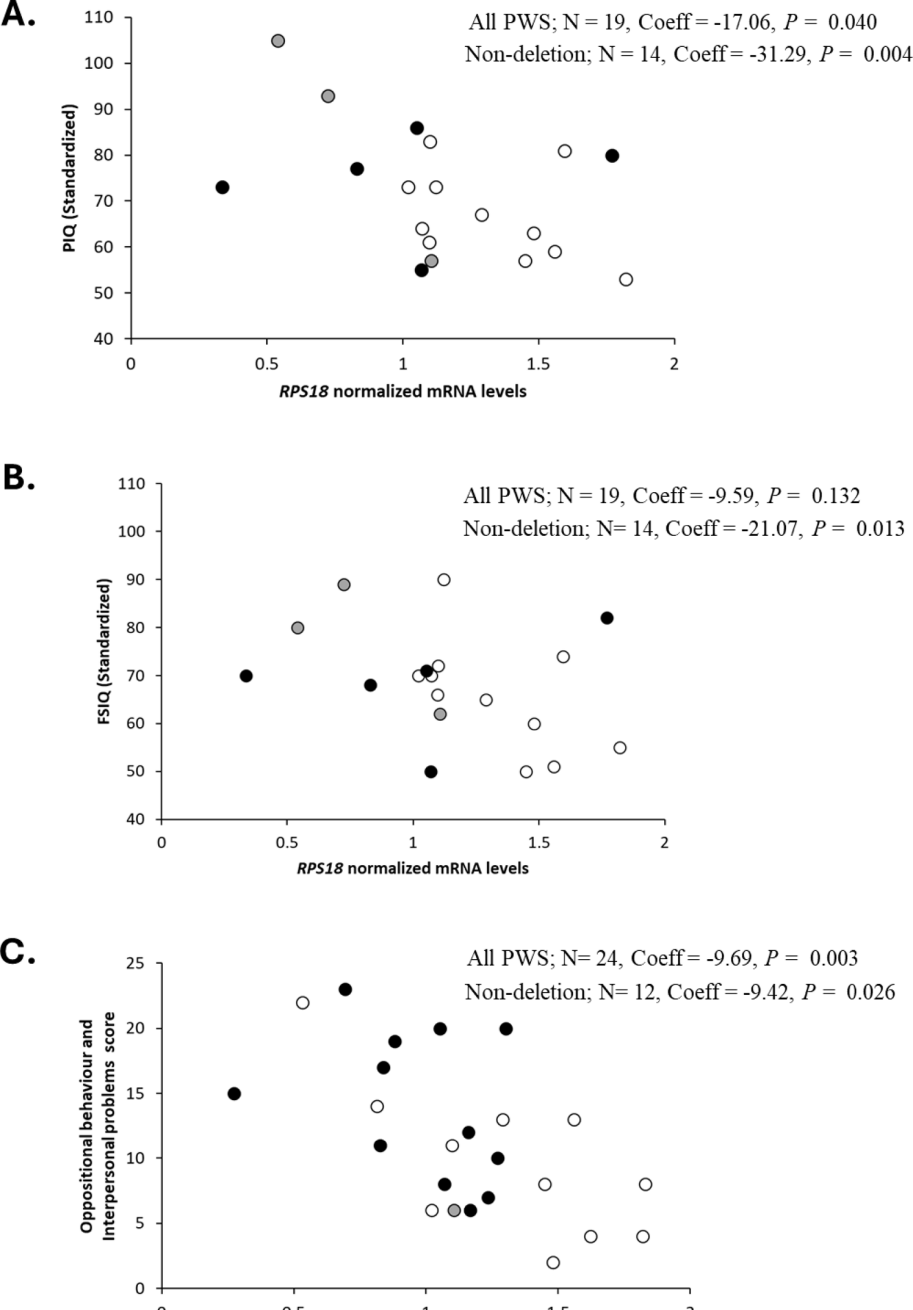
Relationships between *RPS18* mRNA levels assessed using ddPCR in PBMCs of individuals with PWS and: (**A**) Standardized Performance IQ (PIQ) [Non-del *P* = 0.004; Coefficient = -31.29; *N* = 14; All PWS: *P* = 0.040; Coefficient = -17.06; *N* = 19] ; (**B**) Standardised Full-Scale IQ (FSIQ) [Non-del *P* = 0.013; Coefficient = -21.07; *N* = 14; All PWS: *P* = 0.132; Coefficient = -9.598; *N* = 19] (< 13 years of age); (**C**) Oppositional behavior and Interpersonal problems score [Non-del *P* = 0.026; Coefficient = -9.425; *N* = 12; All PWS: *P* = 0.003; Coefficient = -9.696; *N* = 24] children (5 to 12 years) and adults (19 to 45 years). Note: open circles = UPD, grey circles = imprinting center defect; black circles = deletion; Non-del = non-deletion (UPD and ICD combined into one group); All PWS = deletion and non-deletion groups combined into one group, Coeff = Coefficient.

Increased levels of *RPS18* mRNA in PBMCs were associated with decreased intellectual functioning, but not autism features as assessed using ADOS-2 for children with PWS due to non-deletion (< 13 years of age) (Fig. 5A and B; Table 1). The associations with intellectual functioning scores were restricted to performance IQ (PIQ) and full-scale IQ (FSIQ), but not verbal IQ (VIQ). Increased levels of *RPS18* mRNA were also associated with less severe oppositional behaviors in the combined cohort with and without adolescents following correction for FDR (Fig. 5C; Table S5 and Table S6). It is worth noting that for the combined cohort analysis (Table S5) the 2 adolescents included were both outliers on all PWSBQ variables, with the sample size not being sufficiently large to analyze them as a separate group. No association reached significance for *RPS18* mRNA levels and autism features assessed using ADOS-2 for any of the comparisons (Table 1). ADOS-2 measures and PWSBQ scores were not significantly associated with intellectual functioning measures, with ADOS-2 measures also showing no significant associations with any PWSBQ measures (Data S4).

## Discussion

This study identified 54 genes consistently differentially expressed across all cell types of the Brodmann area 9 from all PWS versus control brain tissue comparisons. *RPS18* was the only gene upregulated across all comparisons, for both deletion and non-deletion genetic subtypes of PWS, and the combined deletion and non-deletion PWS group as compared to controls. Our follow-up studies in PBMCs from another cohort found that expression of *RPS18* was also related to intellectual functioning and oppositional behaviors, but not autism features in PWS. Higher levels of *RPS18* mRNA were, on one hand associated with lower intellectual functioning primarily represented by PIQ and FSIQ in children with PWS due to non-deletion, and on the other, with less severe behavioral issues in both children and adults with PWS. While this may appear counterintuitive, it is possible that individuals with higher intellectual functioning would have better comprehension of their conditions, and this could in turn lead to higher levels of frustration and other behavioral issues than in those with lower intellectual functioning.

Interestingly, oppositional behaviors which showed a relationship with *RPS18* mRNA levels, is a subscale of the PWSBQ where parents may be reliant on their child’s ability to communicate their problems. Taking this into consideration, subjects with lower IQ may not have been as readily able to communicate these aspects in an obvious way. Indeed, items relating to oppositional behaviors on the PWSBQ ask whether the child is argumentative, manipulative, and prone to lying, which likely requires a higher level of intellectual functioning to engage in such behaviors. This may be why *RPS18* mRNA levels showed inverse relationships with IQ and oppositional behaviors. Nonetheless, other factors (e.g., medication use, socio-demographics) may moderate the relationships between *RPS18* and clinical outcomes in individuals with PWS. Assessment of the impact of these factors on these observed relationships in participants with PWS would require a larger sample size, and as such, is beyond the scope of this study. Future studies should re-examine these observations in larger cohorts while also considering contribution of psychiatric illness and associated medication use in relation to association between lower *RPS18* and greater behavioral challenges, as previous research suggests involvement of *RPS18* differential expression in complex mental health conditions like schizophrenia^17^.

### *RPS18* upregulation

*RPS18* is ubiquitously expressed across all tissues^18^ encoding a ribosomal protein that is a component of the 40S subunit, which regulates initiation of translation. While its association with symptoms has not been previously described in patients with PWS, *RPS18* has been reported to be upregulated in Parkinson’s disease and colorectal cancer^19,20^. One explanation for upregulation of *RPS18* in our study across all cell types in brain tissues and the relationships between its expression in blood and phenotype may be that upregulation of this gene and related ribosomal pathways is part of a compensation mechanism. This mechanism may counterbalance the loss of PWS-imprinted gene expression at 15q11-q13 across all cell types and tissue types in PWS, including blood^16^. This hypothesis is consistent with a previously proposed ‘compensatory’ natural gene therapy theory where upregulation of *SNRPB* is thought to prevent lethality resulting from *SNRPN* loss in PWS, to maintain functional spliceosomal machinery^21,22^.

Relationships between gene expression changes in PBMCs and PWS symptoms observed in this study may either suggest direct involvement of *RPS18* in the immunological disturbances previously reported in PWS associated with increased plasma chemokine levels^23^ and/or indirect reflection of brain-specific changes consistent between different cell types and tissues, including blood. This is consistent with previous single-cell RNA sequencing studies in PBMCs suggesting that *RPS18* plays an important role in regulation of neutrophil function needed in innate immunity response^24^. Future studies should explore through *in vitro* functional studies and *in vivo* animal studies the consequences of *RPS18* upregulation in different cell types, including to consider potential reversal of cellular and biochemical disturbances associated with loss of PWS-imprinted expression in this condition.

### Ribosomal gene dysregulation in PWS

In addition to *RPS18*, we identified 69 ribosomal DEGs in specific cell types in brain tissue studies from donors with PWS, most upregulated as compared to control brain tissues (Data S3). These findings are consistent with a recent study utilizing embryonic stem cells differentiated into neurons to identify 42 consistently dysregulated genes, including *RPS18* and other ribosomal genes, caused by loss of *SNORD116* from the PWS-imprinted domain^16^. Together these findings are in line with previous studies showing: (i) snoRNAs from the PWS-imprinted domain regulating ribosome biogenesis^25^; and (ii) upregulated ribosomal genes and proteins (including *RPS18)* in PFC tissues of donors affected with idiopathic autism^26^.

Moreover, similar ribosomal upregulation has been observed in maternal immune activation in idiopathic autism and non-neuronal cell types including blood leukocytes linked to neuronal phenotypes^27,28^. This is in line with our finding in PBMCs of donors with PWS, where the level of upregulation was related to PWS severity measures. It is also consistent with the pathophysiological mechanism proposed in a recent bulk RNA-seq study showing that genes associated with inflammatory response were upregulated in PWS hypothalamus^29^.

### Strengths and limitations

One of the major strengths of this study is the utility of snRNA-seq on matched brain tissues from donors with PWS due to deletion and non-deletion genetic subtypes and controls for the largest collection described to date. This was used for discovery of DEGs consistently dysregulated across all cell types examined. Another strength is the utility of an independent validation cohort for gene expression versus phenotype assessments to validate if genes shortlisted in brain tissues are related to the severity of PWS.

Four main limitations of the current study are: (i) the relatively small sample sizes for each of the genetic subtypes of PWS; (ii) over-representation of ICD in relation to UPD or deletion subtypes in gene expression versus phenotype studies; (iii) analysis of only one brain region; (iv) that donors whose brain tissues were analyzed were deceased, where changes detected may not reflect those present in life. Other limitations include: (i) our inability to map full-length RNA isoforms and splicing aberrations due to 3’short-read sequencing employed in this study; (ii) protein-coding DEGs not being confirmed at the protein level; (iii) the follow-up gene expression versus phenotype studies only performed for *RPS18* – but not the other 53 DEGs identified across all cell types of the Brodmann area 9 in this study; and (iv) our analyses in brain tissues being restricted to single-nucleus transcriptomics, and not examining cellular and secreted protein products for shortlisted DEGs. These are important directions for future studies to confirm functional significance of the shortlisted DEGs and their relationships with symptoms reported here.

In this study, deletion, non-deletion and control groups were processed separately, introducing batch effects. Although this experimental design confounds both biological and technical effects, Seurat integration was used to minimize batch effects. Biological variation was still present after integration, shown by cell type-specific clustering across samples and genotypes, also observed in low-dimensional space, indicating that technical variation could be smaller than true biological effect. Future studies should validate these results using orthogonal methods, and experimental designs should randomise samples across sequencing batches to minimise potential batch effects.

Another limitation of this study is that it focused exclusively on six major brain cell types, as further sub-clustering could reduce statistical power due to the low abundance of more specialized cell types. Additionally, the exclusion of endothelial cells due to their low representation may limit the interpretation of vascular contributions, which are relevant to the metabolic aspects of PWS. Future studies should employ more refined sub-clustering to identify additional cell populations present across different brain layers. This would be possible by capturing a greater number of nuclei than were included in this study to provide a deeper understanding of alterations in cellular development and functional states in the brain associated with PWS.

The use of the PWSBQ in children under the age of 12 years may be also considered as a limitation. The PWSBQ was originally developed for children at 12 years and older and has not previously been used in a younger sample. Nonetheless, the scores on the PWSBQ subscales did not show a floor effect, suggesting that the PWSBQ was able to capture PWS-specific behavioral challenges in children aged between 5 and 11 years. Future studies should include other PWS-specific questionnaires such as the PWS Profile^30^ and/or the Hyperphagia Questionnaire for Clinical Trials^31^.

## Conclusion

The study demonstrated the utility of snRNA-seq analysis on post-mortem brain tissues from donors with PWS for discovery of novel candidate genes dysregulated between different cell types in the brain and related to phenotype severity in PWS. Glial cells (primarily oligodendrocytes and microglia) showed the highest number of DEGs and dysregulated pathways compared to all other cell types in the deletion and non-deletion PWS groups, with interneurons being the only cell type showing a statistically significant increase in the percentage of cells in all PWS groups as compared to controls. We found 54 DEGs consistently dysregulated between all cell types in the PFC of donors with PWS. However, *RPS18* was the only protein-coding gene identified to be upregulated in PWS PFC across all comparisons, and for this reason, we followed up this finding with targeted gene expression versus phenotype studies in another cohort. We subsequently found that an increase in *RPS18* mRNA levels in PBMCs was associated with symptoms in individuals living with PWS. These observations in PWS are in line with findings in idiopathic autism of broader ribosomal gene networks upregulation^26^. If confirmed in other brain areas and in larger independent cohorts, these observations may lead to development of new: (i) in vitro human models to assess function on glial cells and interneurons reflecting *in vivo* brain changes in PWS; (ii) prognostic markers and tests utilizing peripheral tissues; and (iii) therapeutic target or repurposing of treatments and/or existing medications used for other indications.

**Figure:**
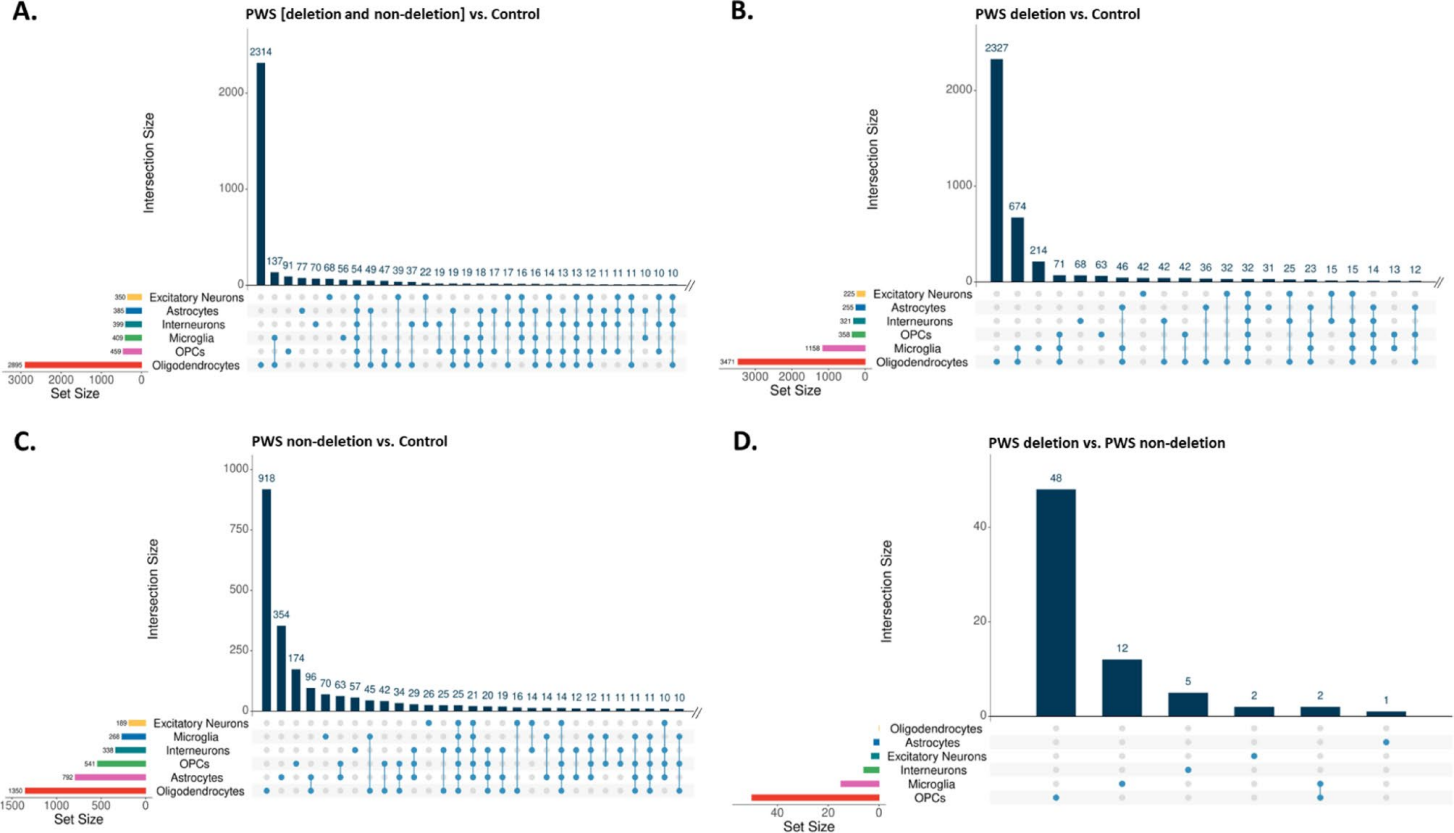
UpSet plot of intersection between the differentially expressed genes (DEGs) in different cell types in the prefrontal cortex of PWS due to deletion, PWS due to a non-deletion and matched controls (*n* = 4 each group). Comparisons between: (**A**) combined PWS group (deletion and non-deletion) and controls; (**B**) deletion PWS and controls; (**C**) non-deletion PWS and controls; (**D**) deletion and non-deletion PWS groups. Note: OPCs = Oligodendrocyte precursor cells. Panels A, B, and C include intersections greater than 10 DEGs.

## Supplementary Information

Below is the link to the electronic supplementary material.

Supplementary Material 1

Supplementary Material 2

Supplementary Material 3

Supplementary Material 4

Supplementary Material 5

## Data Availability

The snRNA-seq data from the PFC tissues can be accessed through NCBI Gene Expression Omnibus, [GSE261461](https:/www.ncbi.nlm.nih.gov/geo/query/acc.cgi? acc=GSE261461). The PBMC gene expression ddPCR and phenotypic outcome measures data from participants with PWS are available on request from the corresponding author. These are not publicly available due to privacy or ethics approval restrictions.
