# Supplementary material for: Transcriptomic signatures in brain and blood related to cognitive and psychiatric phenotypes of Prader–Willi syndrome
Source: Sci Rep. 2025 Dec 24;16:3029. doi: 10.1038/s41598-025-33041-3 (PMC12827965; doi:10.1038/s41598-025-33041-3)
Supplement: Supplementary file 1 — Supplementary Material 1 [file 41598_2025_33041_MOESM1_ESM.docx]

**Supplementary online content**

Shahrokhi S, Baker EK, See M, et al. Transcriptomic signatures in blood and brain related to cognitive and psychiatric phenotypes of Prader-Willi syndrome.

**Figure S1. Chromosomal microarray analysis of single-nucleotide polymorphisms (SNPs) on chromosome 15:19,093,764 -102,531,392 (hg19) to confirm the etiology of 8 brain tissue donors affected with PWS.**

**Figure S2. Pearson correlation between Seurat- and Harmony-derived differential expression results in different cell types in the prefrontal cortex of PWS due to deletion, PWS due to a non-deletion, and matched controls (n = 4 each group)*.***

**Figure S3. Selection of most stably expressed internal control genes in PWS and control groups using the geNorm approach.**

**Table S1. Characteristics of donors and tissues included in brain transcriptomics studies.**

**Table S2. Cell type marker genes used for cluster annotation.**

**Table S3. Primer sequences of candidate internal control genes and resulting product length.**

**Table S4. Relationship between *RPS18* mRNA levels in PBMCS of all participants with PWS, 5 to 45 years and behaviors specific to PWS using the PWS Behavioral Questionnaire (PWSBQ).**

**Table S5. Univariate regression analysis assessing relationship between *RPS18* mRNA levels in PBMCS of children (5 to 12 years) and adults (19 to 45 years) with PWS and behaviors specific to PWS using PWS Behavioral Questionnaire (PWSBQ).**

**Note S1. Cohort characteristics and assessments.**

**Note S2. Sample and data processing for single-nucleus RNA sequencing (snRNA-seq)**

**Note S3. Targeted gene expression analyses.**

**Note S4. Pathway analysis in brain tissues.**

**
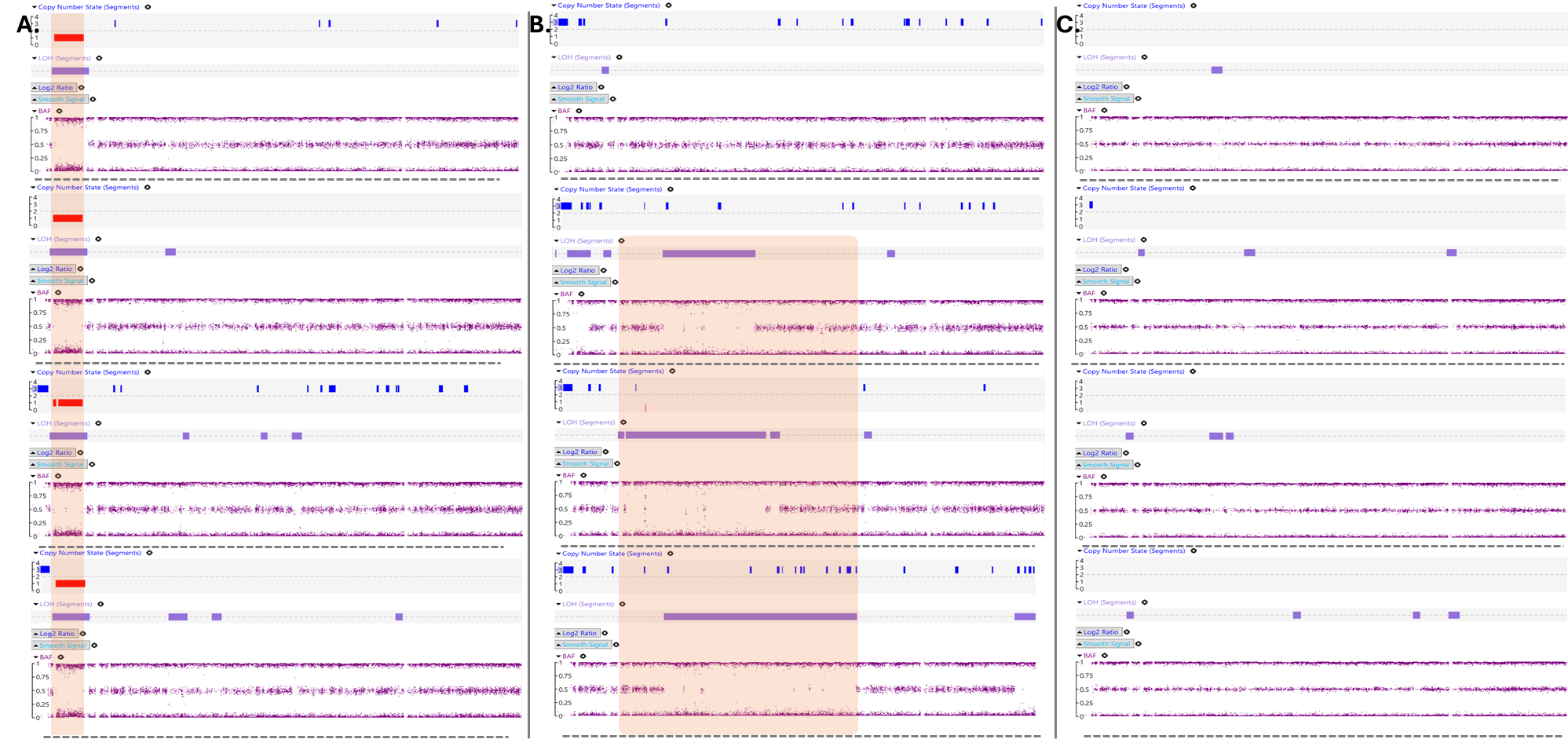
**

**Figure S1. Chromosomal microarray analysis of single-nucleotide polymorphisms (SNPs) on chromosome 15:19,093,764 -102,531,392 (hg19) to confirm the etiology of 8 brain tissue donors affected with PWS.**Each panel represents a SNP genotyping plot from one of the donors in the: (A) deletion, (B) non-deletion, and (C) control groups (n = 4). Note:  Red bars represent copy number loss spanning break points 1 to 3. Purple bars represent loss of heterozygosity regions.

**
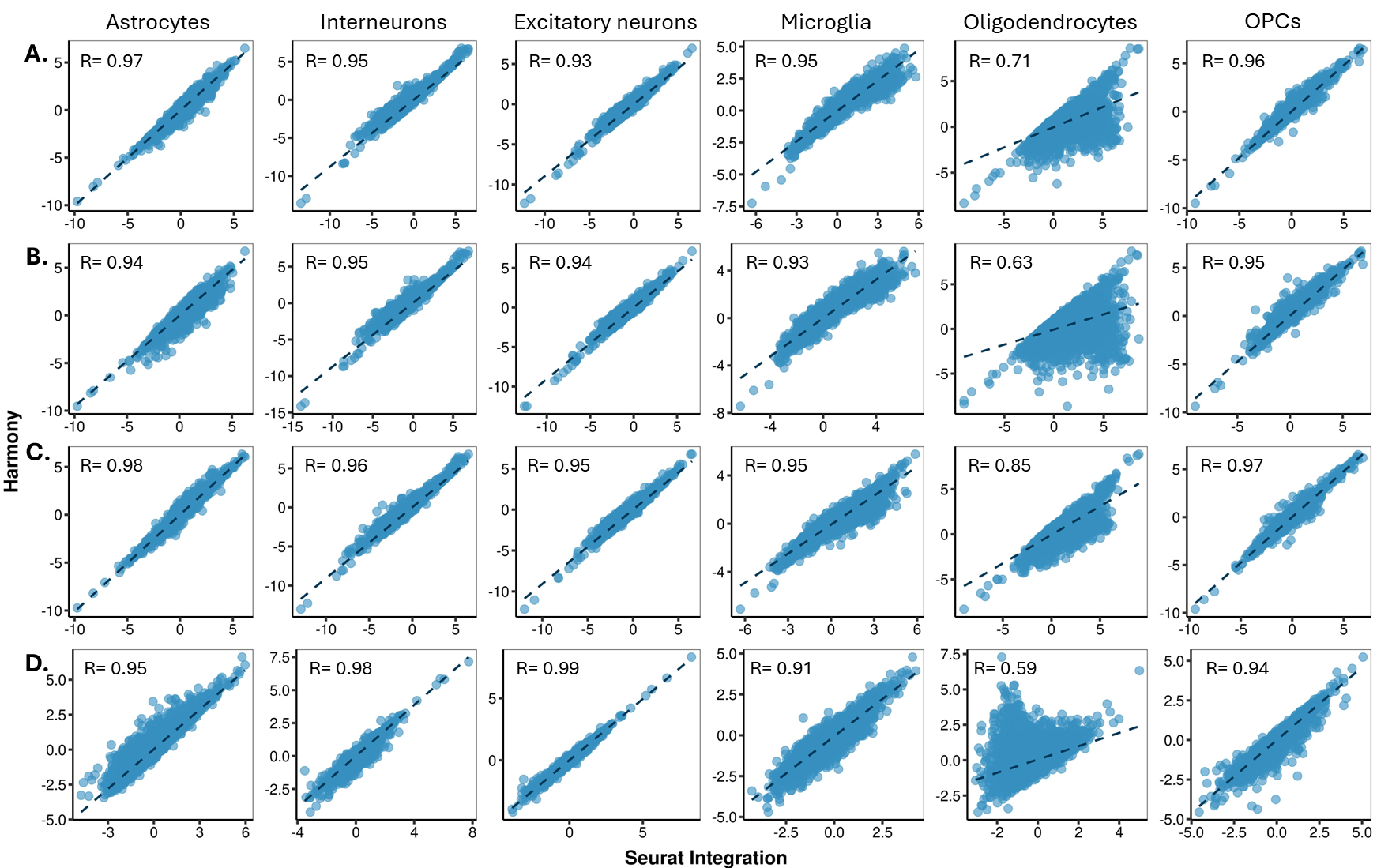
**

**Figure S2: Pearson correlation between Seurat- and Harmony-derived differential expression results in different cell types in the prefrontal cortex of PWS due to deletion, PWS due to a non-deletion, and matched controls (n = 4 each group).** Comparisons between: (A) combined PWS (deletion and non-deletion) and controls; (B) deletion PWS and controls; (C) non-deletion PWS and controls; (D) deletion and non-deletion PWS groups. Note: All correlations P < 2.2e-16.

**
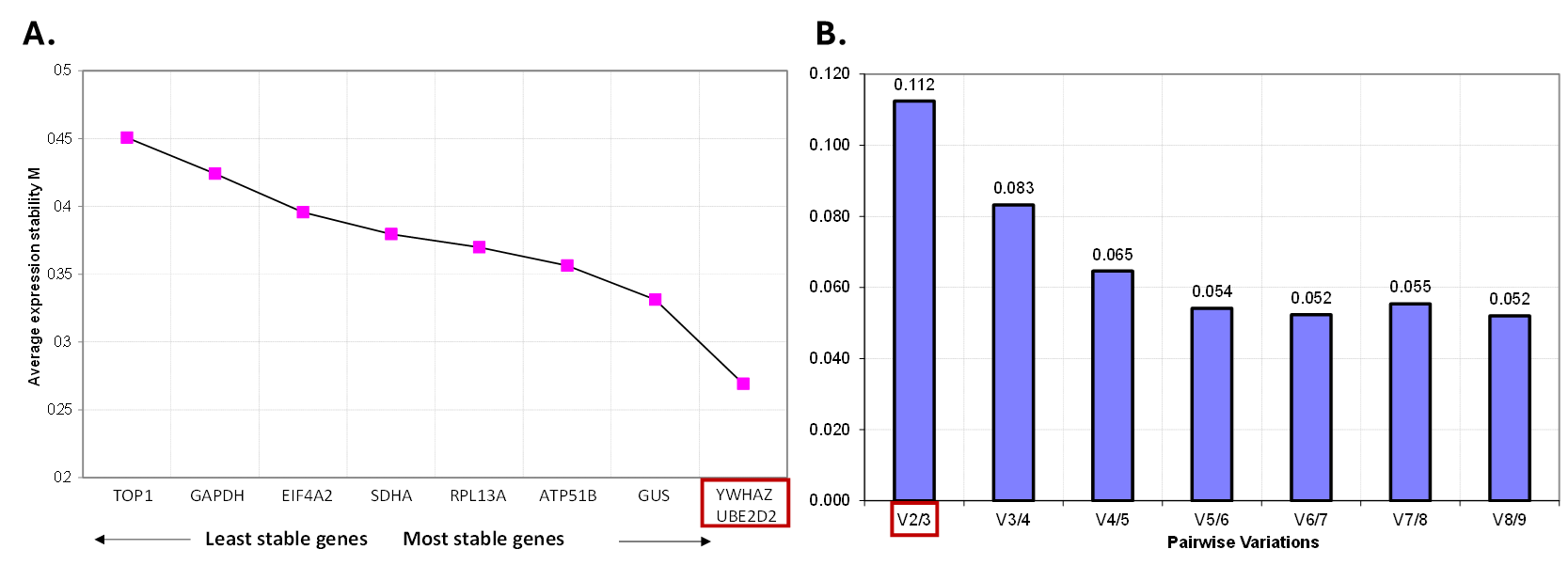
**

**Figure S3. Selection of most stably expressed internal control genes in PWS and control groups using the geNorm approach.** Determining stability of expression for 9 internal control genes using the geNorm approach in peripheral blood mononuclear cells (PBMCs) of 34 individuals affected with PWS and 20 typically developing controls. **A.** Average expression stability M values, with least to most stable ordered from the left to right direction on the X axis. *YWHAZ* and *UBE2D2* were the most stably expressed genes from the panel tested. **B.** Variation in average gene expression stability with sequential addition of each internal control gene to the equation (for calculation of the V score normalization factor).

**Table S1. Characteristics of donors and tissues included in brain transcriptomics studies.**

|  | Control | | |  | Deletion | | |  | Non-deletion | | |  |  |
| --- | --- | --- | --- | --- | --- | --- | --- | --- | --- | --- | --- | --- | --- |
|  | N | Median | IQR |  | N | Median | IQR |  | N | Median | IQR |  | *P* |
| Age (years) | 4 | 37.0 | 16.0 |  | 4 | 37.5 | 16.0 |  | 4 | 35.5 | 10.0 |  | 0.911 |
| Body Mass Index (BMI) | 4 | 29.7 | 15.1 |  | 2 | 36.0 | 16.6 |  | 3 | 45.4 | 33.4 |  | 0.537 |
| Post-Mortem Interval (hours) | 4 | 24.0 | 5.50 |  | 4 | 16.0 | 13.0 |  | 4 | 20.5 | 10.0 |  | 0.551 |
| RNA Integrity Number (RIN) | 4 | 7.05 | 1.00 |  | 4 | 6.15 | 0.50 |  | 4 | 5.80 | 1.15 |  | 0.058 |

Note: *P* = P - value from the comparison of the equality of the median of the three groups using non-parametric Kruskal-Wallis rank test. IQR = interquartile range.

**Table S2. Cell type marker genes used for cluster annotation.**

| **Cell Type** | **Marker genes** |
| --- | --- |
| Microglia | *PTPRC* (1), *ARHGAP25* (2), *ATP8B4* (2) |
| Astrocytes | *SLC1A2* (1), *ADGRV1* (2), *RNF219*-*AS1* (2) |
| Oligodendrocytes | *OLIG1*/2, *ANLN* (2), *PLP1* (3) |
| Oligodendrocyte precursor cells | *PDGFRA* (1) |
| Interneurons | *GAD1* (1) , *GAD2* (1) |
| Excitatory neurons | *SLC17A7* (4), *CUX2* (1) |

**Table S3. Primer sequences of candidate internal control genes and resulting product length.**

| **Gene name** | **Forward primer** | **Reverse primer** | **Product length (bp)** |
| --- | --- | --- | --- |
| *YWHAZ* | 5´-ACTTTTGGTACATTGTGGCTTCAA-3´ | 5´-CCGCCAGGACAAACCAGTAT-3´ | 94 |
| *SDHA* | 5´-TGGGAACAAGAGGGCATCTG-3´ | 5´-CCACCACTGCATCAAATTCATG-3´ | 86 |
| *GAPDH* | 5´-CGCTCTCTGCTCCTCCTGTT-3´ | 5´-CCATGGTGTCTGAGCGATGT-3´ | 81 |
| *ATP5F1B* | 5´-TCACCCAGGCTGGTTCAGA-3´ | 5´-AGTGGCCAGGGTAGGCTGAT-3´ | 80 |
| *RPL13A* | 5´-AGCCTACAAGAAAGTTTGCCTAT-3´ | 5´-TCTTCTTCCGGTAGTGGATCTTGGC-3´ | 126 |
| *EIF4A2* | 5´-AATTCCGGTCAGGGTCAAGTC-3´ | 5´-GCCACACCTTTCCTCCCAAA-3´ | 166 |
| *TOP1* | 5´-GGCGAGTGAATCTAAGGATAATGAA-3´ | 5´-TGGATATCTTAAAGGGTACAGCGAA-3´ | 97 |
| *UBE2D2* | 5´-TGCCTGAGATTGCTCGGATCT-3´ | 5´-TCGCATACTTCTGAGTCCATTCC-3´ | 81 |

**Table S4. Intergroup comparison of brain cell type proportions (excluding oligodendrocytes) between control, deletion, and non-deletion PWS groups.**

|  | **Controls** | | |  | **Non-deletion** | | |  | **Deletion** | | |  | ***P* value for group comparison** | | | | |
| --- | --- | --- | --- | --- | --- | --- | --- | --- | --- | --- | --- | --- | --- | --- | --- | --- | --- |
| **Cell type** | **N** | **Median** | **IQR** |  | **N** | **Median** | **IQR** |  | **N** | **Median** | **IQR** |  | **All PWS vs. control** | **Deletion vs. control** | **Non-deletion**  **vs. control** | **Non-deletion**  **vs. deletion** |  |
| **Excitatory neurons** | 4 | 0.150 | 0.259 |  | 4 | 0.369 | 0.114 |  | 4 | 0.482 | 0.070 |  | 0.0894 | **0.0433** | 0.3865 | **0.0433** |  |
| **Interneurons** | 4 | 0.136 | 0.102 |  | 4 | 0.247 | 0.008 |  | 4 | 0.261 | 0.040 |  | **0.0066** | **0.0209** | **0.0209** | 0.3865 |  |
| **OPCs** | 4 | 0.183 | 0.077 |  | 4 | 0.124 | 0.040 |  | 4 | 0.068 | 0.026 |  | **0.0108** | **0.0209** | **0.0433** | **0.0433** |  |
| **Astrocytes** | 4 | 0.236 | 0.300 |  | 4 | 0.191 | 0.078 |  | 4 | 0.176 | 0.106 |  | 0.7341 | 0.3865 | 0.7728 | 0.5637 |  |
| **Microglia** | 4 | 0.170 | 0.150 |  | 4 | 0.044 | 0.028 |  | 4 | 0.025 | 0.020 |  | **0.0066** | **0.0209** | **0.0209** | 0.0833 |  |
| **Endothelial cells** | 4 | 0.005 | 0.006 |  | 4 | 0.006 | 0.005 |  | 4 | 0.003 | 0.002 |  | 0.8651 | 0.1489 | 0.2482 | 0.1489 |  |

Note: Mann-Whitney test was used for intergroup comparisons, bold = *P* values < 0.05.

**Table S5. Relationship between *RPS18* mRNA levels in PBMCS of all participants with PWS, 5 to 45 years and behaviors specific to PWS using the PWS Behavioral Questionnaire (PWSBQ).**

|  | All (N = 26) | | |  | Non-deletion (N = 13) | | | |  | | Deletion (N = 13) | | |  |
| --- | --- | --- | --- | --- | --- | --- | --- | --- | --- | --- | --- | --- | --- | --- |
|  | Coef | se | *P* |  | Coef | se | *P* |  | | Coef | | se | *P* | |
| Emotion Regulation | -3.176 | 2.247 | 0.170 |  | -6.095 | 3.919 | 0.151 |  | | -1.294 | | 3.670 | 0.731 | |
| Food-seeking | -9.504 | 6.495 | 0.156 |  | -0.839 | 0.972 | 0.406 |  | | -22.34 | | 12.67 | 0.108 | |
| Flexibility | -5.325 | 3.503 | 0.142 |  | -1.053 | 5.536 | 0.853 |  | | -8.499 | | 6.450 | 0.214 | |
| Oppositional behavior and Interpersonal problems | -6.820 | 2.898 | 0.027 |  | -9.425 | 3.610 | 0.026 |  | | -4.610 | | 6.437 | 0.489 | |
| Body related behaviors | -1.578 | 1.609 | 0.336 |  | 0.744 | 1.811 | 0.690 |  | | -2.744 | | 3.351 | 0.430 | |
| Total | -25.23 | 11.25 | **0.034**^1^ |  | -9.435 | 19.22 | 0.633 |  | | -22.28 | | 14.19 | 0.145 | |

Note: All regression analyses were conducted using robust regression, which were not adjusted for age and gender because they did not show significant relationships with the variables included. ^1^Adjustments for FDR did not include total score because it was calculated based on all other variables included in the adjustments.

**Table S6. Univariate regression analysis assessing relationship between *RPS18* mRNA levels in PBMCS of children (5 to 12 years) and adults (19 to 45 years) with PWS and behaviors specific to PWS using PWS Behavioral Questionnaire (PWSBQ).**

|  | All (N = 24) | | |  | Non-deletion (N = 12) | | | |  | | Deletion (N = 12) | | |  |
| --- | --- | --- | --- | --- | --- | --- | --- | --- | --- | --- | --- | --- | --- | --- |
|  | Coef | se | *P* |  | Coef | se | *P* |  | | Coef | | se | *P* | |
| Emotion Regulation | -4.770 | 2.291 | 0.049 |  | -6.095 | 3.919 | 0.151 |  | | -2.804 | | 3.904 | 0.489 | |
| Food-seeking | -12.46 | 6.911 | 0.085 |  | -14.03 | 13.11 | 0.310 |  | | -23.52 | | 14.96 | 0.150 | |
| Flexibility | -6.837 | 3.605 | 0.071 |  | -6.789 | 5.535 | 0.248 |  | | -7.934 | | 7.650 | 0.324 | |
| Oppositional behavior and Interpersonal problems | -9.696 | 2.955 | **0.003**^1^ |  | -9.425 | 3.610 | 0.026 |  | | -7.005 | | 6.689 | 0.320 | |
| Body-related behaviors | -2.602 | 1.903 | 0.185 |  | 1.519 | 2.644 | 0.580 |  | | -3.732 | | 3.711 | 0.338 | |
| Total | -32.12 | 11.49 | **0.011** |  | -5.911 | 24.48 | 0.815 |  | | -49.51 | | 23.02 | 0.060 | |

Note: All regression analyses were conducted using robust regression, which were not adjusted for age and gender because they did not show significant relationships with the variables included. ^1^*P* value (p) < 0.05 after adjusting for multiple testing. Adjustments for FDR did not include total score because it was calculated based on all other variables included in the adjustments.

**Note S1. Cohort characteristics and assessments**

*Cohort Inclusion and Exclusion Criteria*

Inclusion criteria for the brain transcriptomics studies were: (i) confirmatory molecular testing supporting assignment into either deletion, non-deletion, or control groups; and (ii) equal number of matched samples in each comparable group other than sex. While there were 50% males in the control and deletion group, the non-deletion group was composed of 25% males. All confirmatory testing on the brain samples were performed at the Murdoch Children’s Research Institute (MCRI) using *SNURF*-*SNRPN* promoter DNA methylation and real-time PCR *SNRPN* copy number analysis, as previously (5).

All individuals in the deletion group showed a copy number loss on chromosome 15q between breakpoint 1 (BP1) and BP3, as identified by SNP array genotyping, consistent with a type 1 deletion. In the non-deletion group, three of the four individuals showed extended regions of loss of heterozygosity (LOH) on chromosome 15q, with the LOH percentage in this region exceeding 25%, indicative of UPD. One of the 4 donors in the non-deletion group did not have LOH segments at this locus.

While parental testing would be required to distinguish maternal isodisomy from other rare causes, such as ICD, this was not feasible for de-identified samples from donors of these tissues. We thus performed gene expression studies that showed elevated levels of maternally expressed *UBE3A* mRNA as compared to the deletion and control groups. This observation, together with the previously reported prevalence of ICD of 5% (as compared to 40% matUPD), confirms that all donors in the non-deletion group are likely matUPD.

Exclusion criteria were donor’s age, atypical manner of death (excluded if were not natural or accidental, reported by NICHD Brain and Tissue Bank), post-mortem interval (PMI), and body mass index (BMI) parameters that did not match the ranges for the comparator groups, and RNA quality as indicated by the RNA integrity number (RIN <5.5), with specific details provided in Table S1.

Inclusion criteria for the 36 individuals living with PWS (16 deletion and 20 non-deletion) were: (i) diagnosis confirmed using DNA methylation analysis using Methylation-Specific Multiplex Ligation-Dependent Probe Amplification and microarray testing for copy number changes within the 15q11-q13 region, as per standard diagnostic testing protocol (6); and (ii) that verbal participants be able to speak English and non-verbal participants be exposed to English within the home. Having another genetic condition of known clinical significance, or having any significant medical condition (e.g., stroke, head trauma) were the only exclusion criteria.

*Assessments of Intellectual Functioning*

An age-appropriate standardized assessment was completed by all participants with PWS. These included the Mullen Scales of Early Learning (MSEL; children aged under 3 years) (7), the Wechsler Preschool and Primary Scale of Intelligence-3rd Edition (WPPSI-III; children aged 3 years to 6 years, 11 months) (8), the Wechsler Intelligence Scale for Children-4th Edition Australian (WISC-IV; children aged 7 years to 16 years, 11 months) (9), and the Wechsler Adult Intelligence Scale-Fourth Edition (WAIS-IV; individuals aged 17 years and above) (10). Standardized Verbal IQ (VIQ), performance IQ (PIQ), and full-scale IQ (FSIQ) were the primary intellectual functioning variables included in the analyses. As the MSEL does not provide separate VIQ and NVIQ scores, derivative VIQ and NVIQ scores were calculated, as previously (11). Details on calculating derivative VIQ and NVIQ scores are provided elsewhere (11). The Early Learning Composite (ELC) was used as a proxy for FSIQ (12) in the 6 children assessed with the MSEL.

*Assessments of Behavior and Autistic Traits*

The PWS Behavioral Questionnaire (PWSBQ) was used to assess behaviors that are specific to PWS patients (13). The PWSBQ comprises five subscales (Emotion Regulation, Food Seeking, Flexibility, Oppositional Behavior, and Body Related Behaviors) with all subscales, except Body Related Behaviors, summed to create a total score. Parents completed the PWSBQ at the time of the assessment. The PWSBQ was originally developed for children at 12 years and older (13). However, in the current study the PWSBQ was completed by parents of children aged 5 years and above. Children younger than 5 years of age were not included as some of the behaviors assessed by the PWSBQ (e.g. food-seeking and body-related behaviors) may not fully manifest until 5 years of age, with the median age of onset of hyperphagia reported at 4.5 years (quartiles 3–5.25 years) (14).

The Autism Diagnostic Observation Schedule-2^nd^ Edition (15) was used to assess social communication skills and the presence of repetitive and restricted behaviors (autistic traits). The ADOS-2 was used in all children who were cruising/walking and had a mental age of 12 months or greater. The ADOS-2 provides an overall calibrated severity score (CSS), in addition to CSS for the Social Affect (SA CSS) and Repetitive and Restricted Behaviors (RRB CSS) domain.

**Note S2. Sample and data processing for single-nucleus RNA sequencing (snRNA-seq)**

*Processing of Brain Samples*

We have processed matched tissues from PFC / Brodmann area 9 from deceased donors with PWS and controls (Table S1), utilizing ~100 mg per tissue to isolate nuclei for snRNA-seq. Additional ~20 mg per tissue were used to isolate: (i) DNA for single-nucleotide polymorphism (SNP) genotyping for demultiplexing and *SNRPN* methylation and copy number variation (CNV) analyses to confirm PWS aetiology, (ii) RNA to determine RNA Integrity Number (RIN) as part of the inclusion criteria.

*Isolation of Nuclei*

The tissues dissected for isolation of nuclei were incubated for 17 minutes in 2.5 ml of chilled lysis buffer [10 mM Tris-HCl (PH:8) (Sigma-Aldrich; St. Louis, Missouri), 3 mM MgCl2 (Sigma-Aldrich; St. Louis, Missouri), 10 mM NaCl (Sigma-Aldrich; St. Louis, Missouri), IGEPAL^®^CA-630 (Sigma-Aldrich; St. Louis, Missouri), and RNase inhibitor (Promega Corporation; Madison, Wisconsin)]. 2.5 ml of ice-cold 1x Dulbecco’s Phosphate Buffered Saline (dPBS) (Thermo Fisher Scientific; Waltham, Massachusetts) was then added to the lysis buffer, and the tissue was homogenized and filtered through a 30 µm strainer and spun at 2000 x g for 5 minutes at 4˚C.

The supernatant was aspirated and the nuclei pellet resuspended in 400 µl of staining buffer (1x dPBS, 1% UltraPure bovine serum albumin (BSA) (Thermo Fisher Scientific; Waltham, Massachusetts), 0.2 U/µl RNase inhibitor, and 1:10,000 DAPI (Sigma-Aldrich; St. Louis, Missouri), and sorted using flow cytometry with a 100 µm nozzle at 22 psi. The sorted samples were centrifuged at 500 x g for 15 minutes at 4˚C. The supernatant was then removed and the nuclei pellets resuspended in 50 µl of nuclei EZ storage buffer (Sigma-Aldrich; St. Louis, Missouri), counted, and stored at -80˚C.

*RNA Quality Assessments of Brain Tissues*

RNA was extracted from ~10 mg of PFC tissue per donor using the RNeasy kit, as per manufacturer’s instructions (Qiagen, Hilden, Germany), with RNA integrity analysis performed using 4200 TapeStation System, as per manufacturer’s instructions (Agilent Technologies; Santa Clara, California).

*SNP Analysis for Demultiplexing*

DNA was extracted from ~10 mg of PFC tissue per donor using the QIAamp DNA mini kit, as per manufacturer’s instructions (Qiagen, Hilden, Germany). SNP analysis was performed for each sample using Axiom genotyping platform (UK Biobank Axiom Array) as per manufacturer’s instructions (Thermo Fisher Scientific; Waltham, Massachusetts) to allow demultiplexing for snRNA-seq data from pooled samples and confirm the aetiology for each sample.

*Single-Nucleus RNA Sequencing (snRNA-seq)*

Isolated nuclei were further diluted to the same concentration for each sample in each pool and then mixed. A target of 5000 nuclei per sample was applied and a total number of 20,000 nuclei per pool/condition were captured using the 10X genomics platform as previously described (1) and sequenced on a NovaSeq 6000 machine (Illumina, Global) at average depth of 30,000 reads per nucleus. Fastq file generation and gene alignment were performed against the human reference genome (ENSEMBL GRCh38-3.0.0) using CellRanger software (Version 6.0.2) and gene expression matrices were produced for each pool. Demultiplexing of the pooled samples and doublet detection was performed using a combination of four different methods recommended by Demuxafy, a framework containing different tools and pipelines for demultiplexing and doublet detection (16). Doublets were removed if detected as doublets by the majority of the applied methods.

The data were removed from: (i) ambient RNA comprising 8-13% in different pools using SoupX (17); (ii) low-quality and outlier nuclei defined using an absolute dispersion measure known as Median Absolute Deviation (MAD) (18); which includes all nuclei with a high percentage of mitochondrial genes (more than 5 MADs), a high percentage of ribosomal genes (more than 5 MADs), low library size (less than 3 MADs), and a low number of detected genes (less than 3 MADs). After applying all these criteria, the number of nuclei in control, deletion, and non-deletion pools was reduced to 14,754, 15,967, and 12,819, respectively. The ‘cleaned’ snRNA-seq data were normalized using SCTransform v2 (19) to remove variations caused by variation in sequencing depth between samples. To remove the batch effect, data from samples were then integrated using the Seurat integration method (20). The biologically informative features were then selected using runPCA (21) and the first 30 principal components (PCs) were used for noise removal and dimension reduction of the data. Clustering was then performed using SNN method with FindNeighbors and FindClusters functions from the Seurat package version 4.3.0 (21). To annotate the cell types, the expression of known cell type-specific markers was investigated (Table S2). Additionally, the cluster markers identified in our data were compared to the cluster markers reported by Velmeshev et al.(1) to ensure the reliability of cell type identification.

*Cross-method reproducibility of the integration result*

To evaluate the effectiveness of Seurat integration in mitigating batch effects, Harmony was applied as an alternative integration approach, and the resulting differential expression profiles were compared with those derived from Seurat-integrated data. Pearson correlation coefficients exceeded 0.9 across all cell types in every comparison, except for oligodendrocytes, where correlations ranged from 0.59 to 0.85 across the four comparisons, with the non-deletion versus deletion showing the lowest concordance (Fig. S2). The overlap between DEGs identified by the two integration methods was also assessed. A strong overlap was observed for most cell types across all comparisons, with only oligodendrocytes and microglia showing reduced agreement between methods (Data S4). Importantly, *RPS18* was consistently identified as a DEG across all cell types using both methods.

**Note S3. Targeted gene expression analyses**

*Gene Expression Analysis Utilizing ddPCR*

For each assay, 1.0 µl of the primer mix was added to 1X QX200 Evagreen ddPCR SuperMix *(*Bio-Rad Laboratories; Hercules, California*)* and RNAse/DNAse-free water (Qiagen, Hilden, Germany), and then aliquoted into 96-well PCR plates. Two µl of cDNA was then added to the reaction mix. For RPS18 analyses, a premade *TaqMan* Assay (ID: dHsaCPE5190830) was used (Bio-Rad Laboratories; Hercules, California). The assay targets an amplicon with chr6:33243636-33243838 coordinates [on Build hg19] present in most *RPS18* transcripts*,* utilizing a HEX labelled probe with a ZEN/Iowa Black Quencher. Briefly, each 20 μL reaction mixture contained 1.0 μl cDNA, 10 μl ddPCR SuperMix for probes (no dUTP) (Bio-Rad Laboratories; Hercules, California), 0.5 μl *RPS18* primer-*HEX* tagged probe mix, and RNase/DNase-free water. Prepared reactions were run on a Bio-Rad QX200 system (Bio-Rad Laboratories; Hercules, California)*.* Data were analysed using the QX Manager software (Version 2.0), as per manufacturer’s instructions (Bio-Rad Laboratories; Hercules, California).

**Note S4. Pathway analysis in brain tissues**

*Transcriptome-wide Pathway Analysis in Brain Tissues*

We have performed transcriptome-wide gene set enrichment analysis of differentially expressed gene lists identified between groups in PFC studies (Fig. 3). As expected, there were a number of dysregulated pathways shared between deletion and non-deletion groups when compared to controls. These included nervous system development and regulation of cell projection organization (all cell types for both the deletion and non-deletion groups), inorganic ion transmembrane transport and regulation of trans-synaptic signalling (all non-neuronal cell types other than astrocytes for both the deletion and non-deletion groups), regulation of secretion (all non-neuronal cell types other than microglia for both the deletion and non-deletion groups), behavior (all cell types other than excitatory neurons for both the deletion and non-deletion groups), and neuron projections development (all cell types other than astrocytes for both the deletion and non-deletion groups).

The deletion versus control comparisons showed all top 20 pathways to be dysregulated in both oligodendrocytes and microglia (Fig. 3B), while for non-deletion versus control comparisons these were only dysregulated in oligodendrocytes (Fig. 3C). Interestingly, endocannabinoid signalling was one of the most dysregulated pathways in PWS non-deletion versus controls comparisons, but only in non-neuronal cell types (Fig. 3C). Deletion versus non-deletion comparisons showed the least number of DEGs and related pathways affected, as compared to all other comparisons (Fig. 3D), with only OPCs showing 18 pathways dysregulated, and only innate immune response showing dysregulation in both OPCs and microglia, but not in other cell types examined. Moreover, regulation of the innate immune response specific to microglia was the most affected pathway from deletion versus non-deletion comparisons.

**Supplementary References**

1. Velmeshev D, Schirmer L, Jung D, Haeussler M, Perez Y, Mayer S, et al. Single-cell genomics identifies cell type-specific molecular changes in autism. Science. 2019;364(6441):685-9.

2. McKenzie AT, Wang M, Hauberg ME, Fullard JF, Kozlenkov A, Keenan A, et al. Brain Cell Type Specific Gene Expression and Co-expression Network Architectures. Sci Rep. 2018;8(1):8868.

3. Fröhlich AS, Gerstner N, Gagliardi M, Ködel M, Yusupov N, Matosin N, et al. Single-nucleus transcriptomic profiling of human orbitofrontal cortex reveals convergent effects of aging and psychiatric disease. Nature Neuroscience. 2024;27(10):2021-32.

4. Luginbuhl J, Kouno T, Nakano R, Chater TE, Sivaraman DM, Kishima M, et al. Decoding Neuronal Diversification by Multiplexed Single-cell RNA-Seq. Stem Cell Reports. 2021;16(4):810-24.

5. Godler DE, Ling L, Gamage D, Baker EK, Bui M, Field MJ, et al. Feasibility of Screening for Chromosome 15 Imprinting Disorders in 16579 Newborns by Using a Novel Genomic Workflow. JAMA Netw Open. 2022;5(1):e2141911.

6. Butler MG, Hartin SN, Hossain WA, Manzardo AM, Kimonis V, Dykens E, et al. Molecular genetic classification in Prader-Willi syndrome: a multisite cohort study. J Med Genet. 2019;56(3):149-53.

7. Mullen EM. Mullen scales of early learning. Service MAG, editor: Bloomington: AGS Publishing;

; 1995.

8. Wechsler D. Wechsler Preschool and Primary Scale of Intelligence-Australian Standardisation. : Pearson Clinical and Talent Assessment Australia and New Zealand: Sydney; 2004.

9. Wechsler D. Wechsler Intelligence Scale for Children–Fourth Edition: Australian Standardised Edition.: arcourt Assessment: San Antonio, TX; 2005.

10. Wechsler D, editor. The Wechsler Adult Intelligence Scale-Fourth Edition: Administration and Scoring Manual. 4 ed: Orlando: The Psychological Corporation; 2008.

11. Baker EK, Godler DE, Bui M, Hickerton C, Rogers C, Field M, et al. Exploring autism symptoms in an Australian cohort of patients with Prader-Willi and Angelman syndromes. J Neurodev Disord. 2018;10.

12. Bishop SL, Guthrie W, Coffing M, Lord C. Convergent validity of the Mullen Scales of Early Learning and the differential ability scales in children with autism spectrum disorders. Am J Intellect Dev Disabil. 2011;116(5):331-43.

13. Avrahamy H, Pollak Y, Shriki-Tal L, Genstil L, Hirsch HJ, Gross-Tsur V, et al. A disease specific questionnaire for assessing behavior in individuals with Prader-Willi syndrome. Compr Psychiatry. 2015;58:189-97.

14. Miller JL, Lynn CH, Driscoll DC, Goldstone AP, Gold JA, Kimonis V, et al. Nutritional phases in Prader-Willi syndrome. Am J Med Genet A. 2011;155A(5):1040-9.

15. Lord C, Rutter, M., DiLavore, P. C., Risi, S., Gotham, K., and Bishop S. L Autism Diagnostic Observation Schedule, 2nd Edition (ADOS-2): Torrance: Western Psychological Services.; 2012.

16. Neavin. D, Senabouth. A, Lee. J.T.H, Ripoll. A, Consortium. s-e, L F, et al. Demuxafy: Improvement in droplet assignment by integrating multiple single-cell demultiplexing and doublet detection methods. bioRxiv. 2022.

17. Young MD, Behjati S. SoupX removes ambient RNA contamination from droplet-based single-cell RNA sequencing data. Gigascience. 2020;9(12).

18. McCarthy DJ, Campbell KR, Lun AT, Wills QF. Scater: pre-processing, quality control, normalization and visualization of single-cell RNA-seq data in R. Bioinformatics. 2017;33(8):1179-86.

19. Choudhary S, Satija R. Comparison and evaluation of statistical error models for scRNA-seq. Genome Biol. 2022;23(1):27.

20. Stuart T, Butler A, Hoffman P, Hafemeister C, Papalexi E, Mauck WM, 3rd, et al. Comprehensive Integration of Single-Cell Data. Cell. 2019;177(7):1888-902 e21.

21. Satija R, Farrell JA, Gennert D, Schier AF, Regev A. Spatial reconstruction of single-cell gene expression data. Nat Biotechnol. 2015;33(5):495-502.
